# A Comparative Process Mining Analysis of Road Trauma Patient Pathways

**DOI:** 10.3390/ijerph17103426

**Published:** 2020-05-14

**Authors:** Robert Andrews, Moe T. Wynn, Kirsten Vallmuur, Arthur H. M. ter Hofstede, Emma Bosley

**Affiliations:** 1 School of Information Systems, Queensland University of Technology (QUT), Brisbane 4000, Australia; m.wynn@qut.edu.au (M.T.W.); a.terhofstede@qut.edu.au (A.H.M.t.H.); 2 Centre for Healthcare Transformation, Australian Centre for Health Services Innovation (AusHSI), Queensland University of Technology (QUT), Brisbane 4059, Australia; k.vallmuur@qut.edu.au; 3 Jamieson Trauma Institute, Royal Brisbane and Women’s Hospital, Metro North Hospital and Health Service, Brisbane 4029, Australia; 4 Queensland Ambulance Service (QAS), Brisbane 4034, Australia; emma.bosley@ambulance.qld.gov.au

**Keywords:** case study, process mining, data quality, healthcare, ambulance, variant analysis

## Abstract

In this paper we report on key findings and lessons from a process mining case study conducted to analyse transport pathways discovered across the time-critical phase of pre-hospital care for persons involved in road traffic crashes in Queensland (Australia). In this study, a case is defined as being an individual patient’s journey from roadside to definitive care. We describe challenges in constructing an event log from source data provided by emergency services and hospitals, including record linkage (no standard patient identifier), and constructing a unified view of response, retrieval, transport and pre-hospital care from interleaving processes of the individual service providers. We analyse three separate cohorts of patients according to their degree of interaction with Queensland Health’s hospital system (C1: no transport required, C2: transported but no Queensland Health hospital, C3: transported and hospitalisation). Variant analysis and subsequent process modelling show high levels of variance in each cohort resulting from a combination of data collection, data linkage and actual differences in process execution. For Cohort 3, automated process modelling generated ’spaghetti’ models. Expert-guided editing resulted in readable models with acceptable fitness, which were used for process analysis. We also conduct a comparative performance analysis of transport segment based on hospital ‘remoteness’. With regard to the field of process mining, we reach various conclusions including (i) in a complex domain, the current crop of automated process algorithms do not generate readable models, however, (ii) such models provide a starting point for expert-guided editing of models (where the tool allows) which can yield models that have acceptable quality and are readable by domain experts, (iii) process improvement opportunities were largely suggested by domain experts (after reviewing analysis results) rather than being directly derived by process mining tools, meaning that the field needs to become more prescriptive (automated derivation of improvement opportunities).

## 1. Introduction

This paper reports on key findings and lessons learned from a process mining case study conducted to analyse patient journeys during the time-critical phase of pre-hospital care and transport of persons involved and injured in road traffic crashes in Queensland, Australia. Pre-hospital care and transport can be supplied by road services, aero-medical services or a combination of these two services. Both types of service are costly, resource intensive, asset limited and take significant coordination to deploy. Comparing the various transport modes, escort levels, etc. may lead to a better understanding of associated factors contributing to patient outcomes. However, there is limited research internationally examining the retrieval processes for patients from roadside to definitive care, and there has been no research conducted in the Queensland context. Process mining has been successfully applied in the healthcare domain as evident by a recent literature review [1] which discovered 172 articles reporting applications of various process mining techniques in the healthcare domain. In Australia, process mining techniques have been used to conduct a comparative analysis of patients’ care pathways in four South Australian Hospitals [2], and to undertake performance analysis of patients’ length of stay [3]. Insights from these studies and many others [4,5,6] showed potential benefits of utilising process mining techniques in the healthcare domain while also highlighting many challenges associated with varying quality of healthcare data [4,7,8] and the complexity of modelling healthcare processes where variations are the norm rather than the exception. There are several novel and challenging aspects to this study. Firstly, while process mining has been applied to patient flows and care pathways in emergency departments [9,10], to this point, process mining has not been applied to model the retrieval and transport phases of pre-hospital care. Secondly, the retrieval and transport phases include some events with short durations and rapid transitions interspersed with longer duration events. Thirdly, data collection is a mixture of automated and real-time, automated but manually-initiated and manually entered (with sometimes, multiple recording modes being applied to the same activity). Lastly, the retrieval, transport and pre-hospital care (roadside to bedside) process is not a single, end-to-end process, but rather the inter-leaving, and parallel execution of individual service providers’ processes where even points of articulation are not fixed.

The objectives of this study are to discover the range of different care and delivery processes undertaken for road trauma patients from roadside to definitive care, and to conduct comparative performance analyses across various cohorts of patients. Specific research questions include:

RQ 1 What is the range of attendance-retrieval-transport processes?
RQ 2 What is the range of patient and process outcomes?
RQ 3 What specific process and performance variations are observed across different cohorts of patients classified by, (i) transport type provided, and (ii) transport locale (metro, regional and remote locations)?

This paper builds upon the earlier data pre-processing phase of the case study reported in [8,11] in which the project establishment phase and source (event) data quality assessment were discussed in detail. The rest of this paper, in which new work related to the case study, is organised as follows. Section 2 discusses the related work. Section 3 elaborates on the organisational context and key questions of interest. Section 4 describes the source event data collection and log preparation. Section 5 presents findings from two key questions of interest: (1) a comparative analysis of different attendance, retrieval and transport processes involving Queensland Ambulance Services (QAS) and Retrieval Services Queensland (RSQ) and (2) a quantitative assessment of performance. Section 6 presents some options for process improvement resulting from this study and discusses key lessons learned from this study for stakeholders and process mining experts.

## 2. Related Work

**Pre-hospital transport and care:** Pre-hospital retrieval time influences morbidity outcomes in certain trauma patients and understanding the processes which occur in the transportation of trauma patients can inform interventions to improve the timeframes within which patients are transferred to appropriate hospital-based care [12]. While process mining has been applied to the healthcare domain in many case studies [13], there has been only limited attention paid to pre-hospital transport and care. Lamine et al. [14] report on a simulation study of emergency call centre operations in France, and Bruns et al. [15] apply complex event processing (CEP) to improve the availability and accuracy of information for emergency call centre dispatch operators. Badakhshan and Alibabaei [16] apply discovery, conformance checking and performance analysis techniques in a case study involving ambulance services in Iran. Notably, there has been very limited research which examines the intersect of ground and aero-medical retrieval of trauma patients and the influence time-to-appropriate-care has on patient outcomes. Further research is needed to understand the processes when there are multiple service providers [17].

**Process mining and healthcare:** Yang and Su [18] reviewed 37 process mining case studies dealing with clinical pathways. Rojas et al. [5] reviewed 74 papers in which process mining was applied in healthcare. Each paper was characterised according to 11 features including process type, frequently asked questions, analysis perspectives, tools and methodologies. A key finding was that there was a need for improved visualisation and visual analytics techniques and an increased focus on conformance checking. Andrews et al. [3] report on challenges facing process mining analysts when applying process mining in healthcare which arise from the semi-structured nature of healthcare processes and the manner in which healthcare data is collected and stored. The authors discussed challenges associated with data pre-processing and quality assessment, as well as automated process discovery, comparative performance analysis and conformance analysis. Suriadi et al. [19] and Partington et al. [2] describe approaches to performing comparative analysis using process mining techniques in a healthcare setting, i.e., four Australian hospitals treating patients with chest pain. Durojaiye et al. [10] reports on the application of process mining to mapping the in-hospital flow of pediatric trauma patients with the aim of identifying major patient pathways. The study applies the Flexible Heuristic Miner [20] algorithm to model data extracted from the trauma registry of a Level 1 pediatric trauma centre. Similar to our study, Durojaiye et al. [10] describes the complexity of discovered models (28 discovered pathways) despite there being only 8 activities in the model. We note that application of the Flexible Heuristic Miner to data in our study generated extremely complex, unreadable models.

**Process mining tools and techniques for comparative process analysis:** Usually, comparative process analysis requires analysing each cohort of interest separately and then combining the separate analysis outcomes (often in a different tool than that used for the analysis). Such an approach requires many tasks to be conducted manually. In [21], the authors describe ProcessProfiler3D, a process mining tool specifically designed to support simultaneous, side-by-side comparative analysis of different cohorts which uses three-dimensional visualisation to compare the cohorts using a variety of performance metrics. Some process mining tools, e.g., Disco (www.fluxicon.org), provide animation options to visually represent the progress of process cohorts over a model. Inductive visual Miner [22] supports segmenting the log into process cohorts according to case attribute values and provides an animation where tokens (representing the cases in the log) are (i) coloured according to cohort membership and, (ii) traverse a (discovered) model. The tool provides both visual and numeric cohort comparisons.

We argue that process mining in the pre-hospital transport and care setting can provide valuable insights into process performance with particular benefits to process owners from quantifying and understanding both the ‘usual’ or frequently occurring pathways (from roadside to bedside) and the pathways where deviations from clinical guidelines are observed.

### Data Ethics

Release of confidential health information is authorised under the authority of the Director General, Queensland Health. Ethical clearance was obtained from the Royal Brisbane & Women’s Hospital Human Research Ethics Committee (#EC00172).

## 3. Case Study: Organisational Context

**Motivation** The delivery of appropriate and timely prehospital care and transport of seriously injured road trauma patients is critical to patient survival and outcomes. Queensland is unique in regards to the geospatial characteristics and population distribution. Being the second largest state in Australia with more than half the population living outside the metropolitan area, significant challenges are faced by emergency services (ground and air ambulance) in responding to (road) trauma, caring for injured persons at the scene and retrieval and transport of patients to health facilities able to deliver the level of care required by the patients.

Queensland is divided into 15 geographical Local Ambulance Service Network (LASN) areas. There are 296 ambulance response locations across the state, including 229 permanent ambulance locations, 22 hospital-based ambulance locations, 10 airport locations, five field offices, 24 locations with QAS first responders and six locations with honorary volunteers. In addition to road ambulances, aeromedical and helicopter retrieval services are available, coordinated by Retrieval Services Queensland (RSQ) under the governance of the Department of Health, Queensland Government.

In 2016-17 financial year, QAS attended 1.04 million incidents across the state, providing 1.19 million responses. For each incident, a record is created within the Computer Aided Dispatch system to manage and record the ambulance dispatch processes through the Operations Centres. For each case where paramedics provide patient assessment and management, an electronic Ambulance Report Form (eARF) is completed containing patient demographic, clinical, scene and management information. In 2016/17 financial year, there were 1,394,096 admitted patient episodes and 12,927,275 non-admitted occasions of service for all diagnoses (https://www.health.qld.gov.au/__data/assets/excel_doc/0028/366616/activity.xls). Qld hospitals had an acute care bed capacity of 11,881 in the same time period with approximately 231 public and private hospitals (https://www.aihw.gov.au/getmedia/d4e53b39-4718-4c81-ba90-b412236961c5/21032.pdf.aspx?inline=true) servicing a population of 4.9 million people in that time period.

Road traffic crashes frequently involve multiple vehicles, multiple passengers (patients), and multiple emergency services response units. Further, any response unit may attend/treat more than one injured person, and any injured person may be attended/treated/transported by more than one response unit. Thus, there are many possible notions of case that can be constructed around road traffic crashes. In this study, we take a case as being an individual patient’s journey from roadside to definitive care.

**Data sources** At the time of this study, a significant challenge in reviewing retrieval processes at the level of individual patient journeys was the lack of data integration between ground-based and aero-medical service providers and state-run health facilities (emergency departments and hospitals). That is, linking patient data collected by the individual emergency services, emergency departments and hospitals is not automated. The Data Linkage Unit within Queensland Health is now responsible for routinely linking both QAS and RSQ data with Qld Health’s existing hospital databases (including emergency department presentation data, admitted patient data and deaths data) (DLU linkage process in https://www.health.qld.gov.au/__data/assets/pdf_file/0030/150798/qlddatalinkframework.pdf) to facilitate the identification of patients that have been attended to by both QAS and RSQ.

## 4. Data Collection and Event Log Preparation

In constructing the event data for this study, data from the following sources was linked by Qld Health’s Statistical Services Branch Data Linkage Unit: (1) Queensland Ambulance Service (QAS), (2) Retrieval Services Queensland (RSQ), (3) Emergency Department Collection (EDC), (4) Queensland Hospital Admitted Patient Collection (QHAPDC) and (5) Births, Deaths and Marriages Data (BDM). As patient journeys were the analysis unit for this study, linkage resulted in a patient identifier being added to each record of each individual source data set. It is worth noting that the event log used in the study was created from event data collected from multiple emergency services providers, with each provider having their own processes for conducting their specialist service. A particular challenge in constructing the event log was blending event data from interleaving processes of the participating service providers with multiple points of articulation between underlying processes.

### 4.1. Data Sources and Recording Practices

Data requested for this study were restricted to those road traffic crashes occurring in the most recent stable years available (i.e., July 2015–June 2017).

QAS uses two separate systems to gather incident, waypoint and clinical intervention data; one system collects vehicle-related data and the other collects patient-related data. Ground-based QAS vehicles in urban and inner-regional areas are fitted with a mobile data collection unit which records vehicle-related waypoint information when an ambulance officer presses a button. However, (i) not all vehicles are fitted with mobile data collection units, (ii) sometimes vehicles are in areas where mobile reception is not possible and (iii) sometimes the underlying information system is off-line. QAS patient-related data is generally not automatically recorded, i.e., the data is firstly recorded by paramedics (noted temporarily while the paramedic is with the patient) and then later transcribed into a digital information system.

In general, aero-medical waypoint times and details of clinical interventions are also recorded manually and transcribed, some time after the actual event, into an information system. Individual aero-medical service providers send data extracts to RSQ on a monthly basis. RSQ, standardises and consolidates these data extracts on a monthly basis and makes the consolidated data available to Qld Health.

Where patients were transported to an emergency care facility that reports to Qld Health, details of emergency presentations are recorded in Qld Health’s Emergency Department Collection (EDC). Where the patient was admitted to a hospital that reports to Qld Health, admission details are recorded in the Queensland Hospital Admitted Patients Data Collection (QHAPDC). Where a transported patient is delivered to a non-reporting healthcare facility, e.g., a private medical practice, no details of post-transport care were available.

Case information from each data source was linked to records in the EDC, QHAPDC and Births-Deaths-Marriages systems and an identifier was assigned by the Data Linkage Unit to indicate which records belong to the same event (road trauma patient transport).

### 4.2. Event Log Preparation

In this study, a case was taken to be an individual patient pathway (from roadside to bedside). Thus, for any road traffic crash (RTC), multiple cases may be created (as any RTC may involve multiple injured persons). Data from QAS and RSQ was provided in tabular (Excel) format where each column represented an attribute of the attendance/transport (e.g., incident identifier, electronic accident report form identifier and patient and vehicle waypoint times). Each record in the QAS and RSQ data set contained multiple waypoints. Similarly, data from Qld Health (EDC and QHAPDC data sets) was provided in tabular (Excel) format and included patient identifier and details of the hospital interaction (whether the interaction was in the emergency department or as an admitted patient). Each EDC and QHAPDC record referred to a single hospital interaction and included start and end times for the interaction. (Note that any patient may have multiple interactions recorded in the EDC and QHAPDC data sets.) Cases were generated by linking the record sets using common patient identifier and timestamp proximity. This was to account for those patients who were involved in more than one RTC during the period covered by the study.

**Figure 1:**
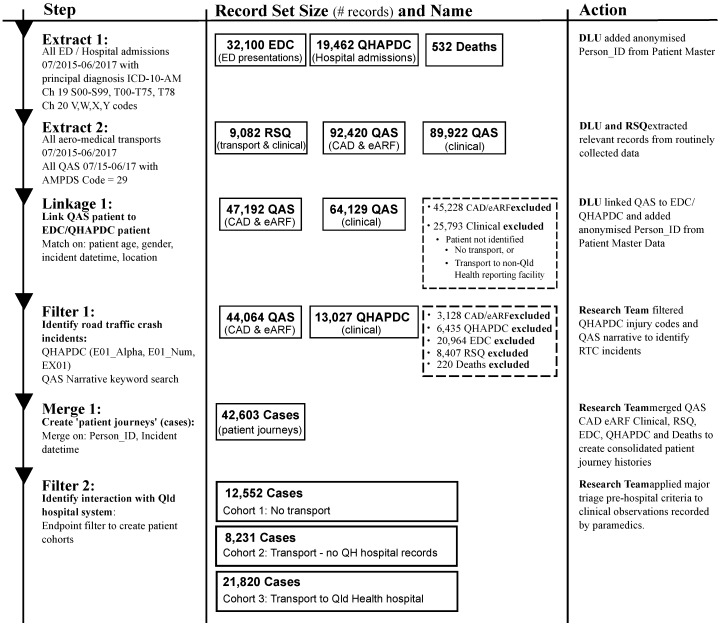
Event log generation—from source data to main analysis cohorts.

**Figure 2:**
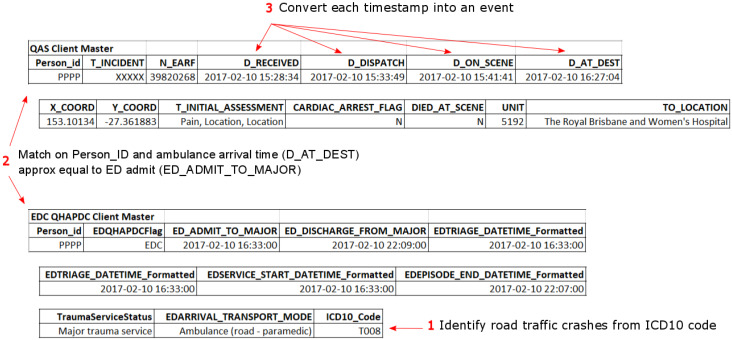
Sample data from Queensland Ambulance Services (QAS) and Emergency Department Collection (EDC) with data attributes—log generation.

**Figure 3:**
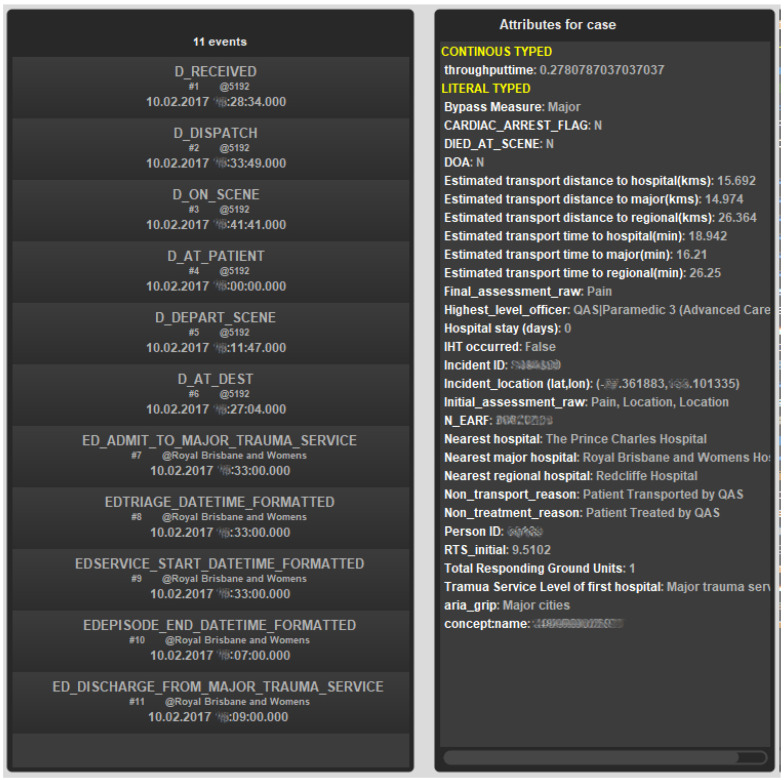
Example case record—with case attributes (identifiers deliberately obfuscated).

It was also necessary to filter the resulting linked record set to remove incidents that did not represent road traffic crashes. The QAS data extract included transport related incidents (AMPDS code = 29). As such this extract included a number of incidents relating to boats or jet-skis, BMX bikes, etc. The record set was filtered to include only incidents representing road traffic crashes (RTCs) by searching for keywords in the T_NARRATIVE field commonly used by paramedics when attending RTCs. To be inclusive, the ICD-10-AM principal diagnosis codes of the QHAPDC data set were searched to identify injuries related to road traffic crashes. Finally, the patient journeys were split into three separate cohorts for analysis based on whether the patient required transport, and if so, did the transport result in hospitalisation. Figure 1 gives details of the numbers of records from each data source that was used in preparation of the study data and the steps involved in generating the event logs used in the process mining analysis. Figure 2 is an example of related records from the QAS and ED data sets which shows (1) how hospital presentations related to road traffic crashes were identified, (2) how QAS records were matched with ED records (same approach for QHAPDC and RSQ records) and (3) how events were created from waypoint timestamps. The corresponding case from the event log is illustrated in Figure 3.

A summary of the event log is shown in Table 1 and a dictionary of the meanings associated with activity labels is given in the Appendix A (see Table A1). It should be noted that we have considered the patient journey to include all emergency services and relevant hospital events from the initial call to the QAS emergency call centre up to a maximum of eight days following the emergency call. We took relevant hospital events to those EDC or QHAPDC events with less than 24 h between the completion of one and the beginning of the next activity up to a maximum of 8 days. An extract of the log (single case from Cohort 3) is shown in Figure 3. The extract shows events and case attributes. For this case, the patient was transported (by QAS) to a Qld Health Facility (Royal Brisbane and Women’s Hospital). Even though the patient met criteria for transport to a major trauma service (Bypass Measure = Major) and the patient was transported to a major trauma service (trauma Service Level of first hospital = Major trauma service), the Revised Trauma Score (RTS = 9.5102) being towards the upper end of the scale, is associated with a better chance of of survival. The patient was not admitted to the hospital (Hospital stay (days) = 0) and was treated, then discharged from the emergency department.

## 5. Case Study Findings

In this section we (i) show how we addressed each of the research questions, (ii) describe our approach and (iii) the tools we used to answer the questions and generate results.

In this case study we used techniques/plug-ins from the open-source ProM (www.promtools.org) framework for all process mining analyses. In particular, we selected the Inductive visual Miner [22] as a tool that is suitable for discovery, conformance and (comparative) performance analyses. We found this tool to have excellent data filtering capabilities and was robust enough to deal with complex models plus it supports direct editing of models.

### 5.1. **RQ 1** What Is the Range of Attendance-Retrieval-Transport Processes?

During the exploratory phase of the case study, we observed different process behaviours (e.g., 2863 trace variants) among the 42,603 cases in the data set. To investigate **RQ 1** we conduct an endpoint analysis in which the log is split into three cohorts based on their end destination event as follows:

- Cohort 1: Attend but no transport—(29.5% of log)
- Cohort 2: Transport, no link to a Qld Health facility—(19.3% of log)
- Cohort 3: Transport to Qld Health facility—(51.2% of log)

Cases belonging to Cohort 1 were characterised by having (i) only events with QAS as org:group, and (ii) no event with activity label as D_AT_DEST, i.e., no record of an ambulance arriving at some destination with a patient on-board.

Cases belonging to Cohort 2 were characterised by having (i) no records with EDC or QHAPDC as org:group, and (ii) at least one event with activity label as D_AT_DEST, i.e., record of an ambulance arriving at some destination with a patient on-board.

Cases belonging to Cohort 3 were characterised by having (i) at least one event with EDC or QHAPDC as org:group.

This segmentation of cases was selected as it represents varying levels of care provided to patients involved in road traffic crashes. At the least severe end of the injury spectrum (Cohort 1) are those patients that require no transport (and sometimes, no treatment). These patients were involved in an accident to which at least one ambulance was dispatched. Records in this cohort are characterised by having an eARF number but with an empty Person_ID value, or, both eARF number and person_ID are empty. As Cohort 1 consists of only the process fragment from emergency call received to on scene (or with patient), there are only 4 distinct events recorded in this cohort, and 43 process variants.

Cohort 2 represents cases where transport is provided to a patient, but based on the transport destination, the injuries received are minor. In general, for Cohort 2, transport destinations include local medical practices, aged care facilities and other private (non-reporting) medical facilities. (There are a number of cases where the destination is a Qld Health reporting facility, but there are no matching EDC or QHAPDC records for the incident/patient).

Cohort 3 represents cases where transport is provided to a Qld Health reporting hospital. For patients in Cohort 3 at least one ED presentation is recorded. Patients in this cohort may also have at least one hospital admission. Inter-hospital transfers may also be included for these patients indicating that injuries received in the accident require treatment not available at the first hospital to which the patient was transported.

**Figure 4:**
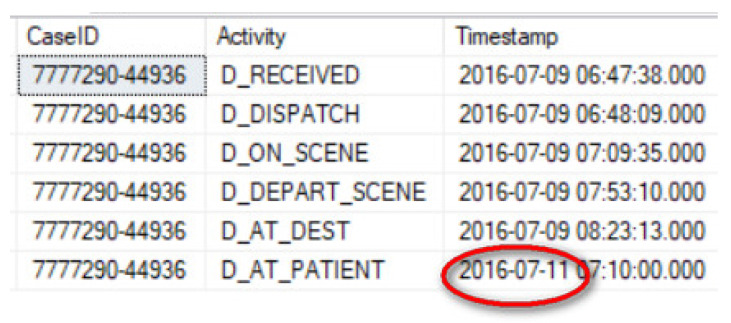
Exceptional case duration due to event timestamp data entry error.

It is worth noting from Table 2 that the maximum case duration for Cohorts 1 and 2 seem extraordinarily long for the type of cases included in each cohort. In each cohort, the case with the maximum duration includes a data error that affects the case duration. For instance, as shown in Figure 4 the “day” value for the D_AT_PATIENT event has been incorrectly entered as “11” instead of “9” resulting in a case duration of 2 days 22 min instead of 96 min.

#### 5.1.1. Cohort 1: Attend But No Transport

**Figure 5:**
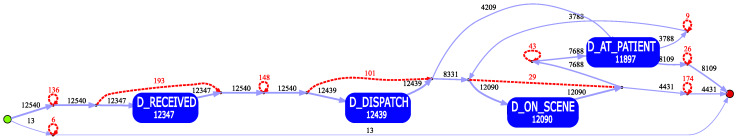
Process model of Cohort 1—no transport.

The (automatically) discovered process model for this cohort (from the Direct Follows Inductive visual Miner [23]) is shown in Figure 5 where after an emergency call is received, an ambulance is dispatched, the ambulance arrives on the scene or/and at the patient and no other event has been recorded for these cases. Cohort 1 comprised 40 trace variants with the median and mean case duration as 10.1 and 16.1 min respectively.

The discovered model shows that, following the D_DISPATCH event, the D_ON_SCENE and D_AT_PATIENT events may be executed in either order (where in actuality, the ambulance must arrive on the scene before the paramedics attend the patient). Investigation revealed a data quality issue associated with the timestamps of these two events, i.e., D_AT_PATIENT events are recorded at the minute level and D_ON_SCENE events at the second level. Thus where the two events actually occurred within the same minute, the D_AT_PATIENT event will be considered to have happened before the D_ON_SCENE event. For example: D_ON_SCENE = ‘2017-03-20 09:45:15’ and (actual) D_AT_PATIENT = ‘2017-03-20 09:45:55’ will result in (recorded) D_AT_PATIENT = ‘2017-03-20 09:45:00’ thus apparently occurring before D_ON_SCENE.

#### 5.1.2. Cohort 2: Transport to Non-Qld Health Facility

**Figure 6:**
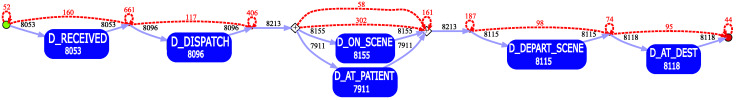
Process model of Cohort 2.

**Figure 7:**
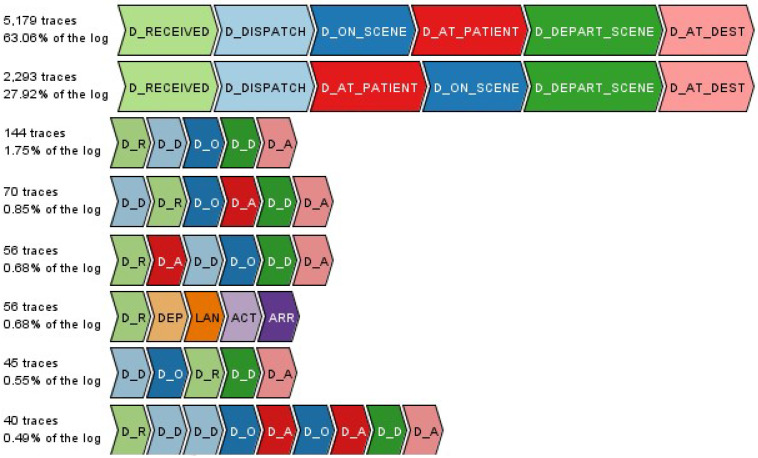
Cohort 2—most frequent trace variants.

The process model of this cohort can be seen in Figure 6 which depicts that after a call for ambulance is received, an ambulance is dispatched, it arrives on the scene/at the patient, the patient is then loaded or triaged at the scene, the ambulance then departs from the scene and finally arrives at the destination recorded as the last activity D_AT_DESTINATION. Cohort 2 comprised 122 trace variants with median and mean case duration as 53.7 and 61.3 min, respectively. Figure 7 illustrates some of this cohort’s trace variants.

The multiple trace variants of this cohort as well as observed deviations in the process model showcase a number of characteristics. First, the interchange of D_ON_SCENE and D_AT_PATIENT activities within the trace variants, as well as D_LOADED and D_DEPART_SCENE can be considered as data quality and accuracy issues which are discussed in Section 5.1.1. Some of the activities also are skipped and not performed (e.g., in some circumstances the patient is assessed on scene and is not loaded onto the ambulance). Consequently, in this instance the D_LOADED activity has not occurred/is not recorded. According to the process owner, possible reasons for these trace variants include patients not admitted to a Qld hospital facility, interchange of transport happened (e.g., the ambulance is called to a regional area, transports patient to a local school, the only place that helicopter can land to transport the patient to a trauma service), or flexibility in data recording protocols. Nevertheless, as the two most frequently occurring trace variants account for 91% of cases in this cohort (with the only difference between trace variants being the order of the D_ON_SCENE and D_AT_PATIENT activities), it can be concluded that this cohort exhibits strong conformity to expected process behaviour.

#### 5.1.3. Cohort 3: Transport to a Qld Health Facility

Queensland Health operates 5 hospitals able to provide major trauma services. Of these, 4 are located in the south-east of the state (major population area) with 1 major trauma service hospital located in the north-east of the state. There are 14 regional trauma services located in primarily coastal areas with reasonably high populations. Only 1 regional trauma service is located in the west of the state. There are approximately 200 other hospital and healthcare facilities operated by Queensland Health throughout the state.

**Figure 8:**
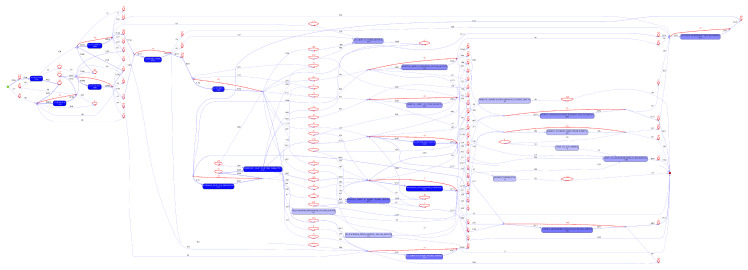
Automatically discovered process model of Cohort 3—80% path abstraction.

The automatically discovered process model from the Inductive visual Miner is shown in Figure 8. The automatically discovered process model showed high fitness (0.96), and reveals the interleaving of processes/activities provided by the respective emergency services. The model reflects the fact that points of articulation between the emergency services can happen at multiple points in the patient journey. Process experts however, found the model complex and hard to read. In particular, it was difficult to follow patient journeys from roadside to the various trauma service levels. Further, on using the modelling tool’s options for reducing complexity (limiting activities and paths considered), the activities associated with aero-medical transport were immediately filtered out of the model and sequential paths (which showed options for interleaving of activities over the different emergency services) were reduced to optional concurrent execution of the individual activities.

Accordingly, the model was manually edited (Inductive visual Miner supports real-time model editing) with an expert from each of QAS and RSQ providing domain and process knowledge, and guidance. The resulting model had somewhat lower average trace fitness (0.91), but was easier to read, and, in the view of the process owners, provided a better view of process pathways (patient journey options) than the automatically discovered model.

**Figure 9:**
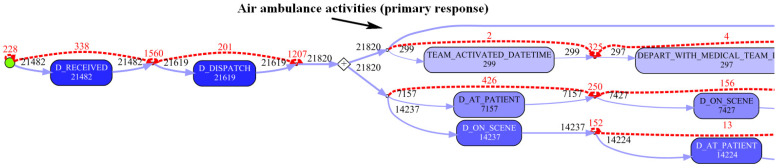
Edited process map fragment (Cohort 3)—air ambulance primary response.

**Figure 10:**
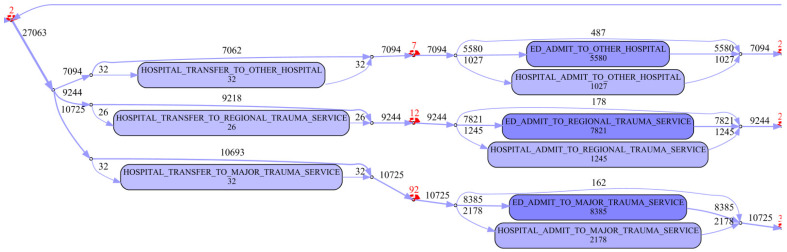
Edited process map fragment (Cohort 3)—trauma service level discharge or transfer to another hospital.

The edited model makes it clear that, the vast majority of cases (individual patient journeys) involve a single ground-based ambulance attending and transporting the patient. Where an air ambulance is required, it is frequently used as primary response, i.e., involved in transport from the scene (see Figure 9). The transport (by ground or air) is to a health facility categorised according to its level of trauma service (major, regional or other). The attending paramedics make a choice as to destination facility depending on incident and hospital location (thus determining travel time), patient status, injury severity, and injury pattern. The edited model shows that for each of the three trauma service levels, approximately 95% of patients transported are ‘handed over’ to the facility’s emergency department (slightly higher for regional trauma service (97%), with the remainder being directly admitted to the hospital (see Figure 10). Of the patients initially presenting to the emergency departments, approximately 18% (slightly higher, 23% for major trauma services) progress to a hospital admission.

**Figure 11:**
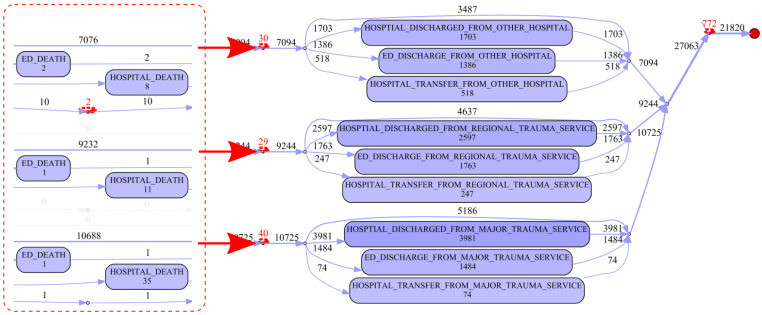
Edited process map fragments (Cohort 3)—trauma service death/discharge/transfer.

Figure 11 is a process fragment showing the death/discharge/transfer options from the various trauma service levels.

A limitation of the edited model was that some of the infrequent pathways (which were of interest to the researchers and domain experts) were not modelled and appeared then as ‘deviations’ on the model. As the discovered model also had significant deviations, it was not apparent which were real deviations and which were artefacts of alignment.

Despite there being only 5 major trauma service facilities in the state, the model shows 40% of all road traffic crash patients transported by emergency services are handled by major trauma service hospitals with the 14 regional trauma services accounting for 35% of transported patients and the 200 other hospitals and health facilities handling the remaining 25% of transported patients. Further investigation is required to determine if this is reflective of population distribution in the state.

### 5.2. **RQ 2** What Is the Range of Patient and Process Outcomes?

We use the endpoint analysis to address **RQ 2** based on some coarse measures of patient outcomes relating to death/discharge. For process outcomes we report on last recorded event and interpret this in light of process executions. We also consider variations (as shown in the process model).

The emergency services retrieval/transport/pre-hospital care process investigated in this study differs from many other business processes in that there is no simple measure for success of the process. If we consider a loan application process, for instance, process outcomes include early termination of the application, or the process completes with a decision as to the success or failure of the application. Close analysis of both the automatically discovered and edited process models show the process may terminate at almost every event (as the models afford the option to ‘skip’ events to process end). Table 3 summarises process outcomes from the point of view of last events per case.

Endpoint analysis for Cohort 1 (dispatches where the patient was not transported by QAS) shows QAS paramedics recorded ON_SCENE as the final event in 4398 cases. However, as described in Section 5.1.1, data quality issues impact on the ordering of these two events. In fact, only 533 cases do not have an AT_PATIENT event recorded in the case.

Reasons for there being no patient transported include: (i) 7222 cases where paramedics directly recorded that “transport was not required”, (ii) 566 cases where transport occurred by private or other means, (iii) 357 cases where the ambulance was stand-by only, (iv) 296 cases where the ambulance was cancelled/recalled, (v) 281 cases where the patient was dead on arrival or died at scene and (vi) a small number of cases where no patient was found at the scene when the ambulance arrived.

Endpoint analysis for Cohort 2 (patient transport but no hospital records) reveals the possibility for data extraction or linkage errors with a small number of cases (1.8% of the cohort) recorded as being transported to a Qld Health facility, but there being no Qld Health ED or hospital records for the patient.

Endpoint analysis for Cohort 3 shows that all patients were transported to a Qld Health facility and arrived alive. A small number (61 patients) were recorded as dying within the scope of the period covered by the event log, with the overwhelming majority (98%) of patients recorded as being discharged from the hospital system within the scope of the event log.

**Figure 12:**
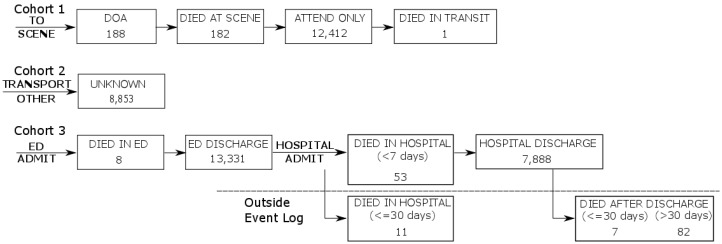
Model of patient outcomes by cohort. Nodes represent patient outcomes, i.e., points of exit from the process. Node numbers show the number of patients with that outcome.

From a patient outcome perspective, we can use only coarse measures including mortality (at various stages of the process including 30 day survival), discharge from ED and discharge from hospital. Figure 12 shows a view of the combined process focusing on the measures of patient outcomes able to be derived from the recorded event data. It can be observed that apart from 183 cases (out of 42,603 cases in the log) where patients died at the scene or in transit, patients were delivered to emergency care alive.

### 5.3. **RQ 3** What Specific Process and Performance Variations Are Observed across Incidents Occurring in Metro, Regional and Remote Locations?

In this section we report on results of a comparative process and performance analysis in which Cohort 3 was further sub-divided according to initial transport destination. Cases were tagged as ‘metro’, ’regional’ or ‘remote’ using the Accessibility Remoteness Index of Australia (ARIA) (https://www1.health.gov.au/internet/publications/publishing.nsf/Content/ARIA-Review-Report-2011~ARIA-Review-Report-2011-2~ARIA-Review-Report-2011-2-2-3) data attribute associated with each Qld Health hospital/facility. The log was re-coded to represent major transport and care segments:

- Primary transport: dispatch to pick up location (which may be scene of incident or intermediate point like an aerodrome) → reaching destination (which may be hospital or handover point to other crew)
- Hospital encounter(s): hospital/ED admit → (optional) inter-hospital transfer → discharge

Activities relating to primary transport in the re-coded log show transport modality (ground or air ambulance). Inter-hospital transfer (hospital encounters section) were always by air ambulance. The process model was manually drawn (and visualised in Inductive visual Miner) but allows for (i) multiple primary transport segments, (ii) multiple hospital encounters (including intra- and inter-hospital transfers) and shows high (0.974) trace fitness. The model showed that the most frequently occurring pathway is from scene of incident to hospital with patient retrieval and transport involving only a single ambulance (20,651 cases out of 21,820 cases). The model also shows that, most often, patients stay in the first hospital to which they are transported (only 339 patients required inter-hospital transfer). (Note that this may involve an ED presentation and a subsequent admission to the same hospital.)

The process model showed that (performance) bottlenecks, i.e., longest duration activities occur for the events relating to inter-hospital transfer (Request–Depart) and Hospital Transfer-Out. This makes sense in light of (i) the interleaving (RSQ and hospital processes) with RSQ frequently being advised (well) in advance of an impending inter-hospital transfer, and (ii) the time required to prepare an aircraft for flight and activate the medical team required for the flight.

**Figure 13:**
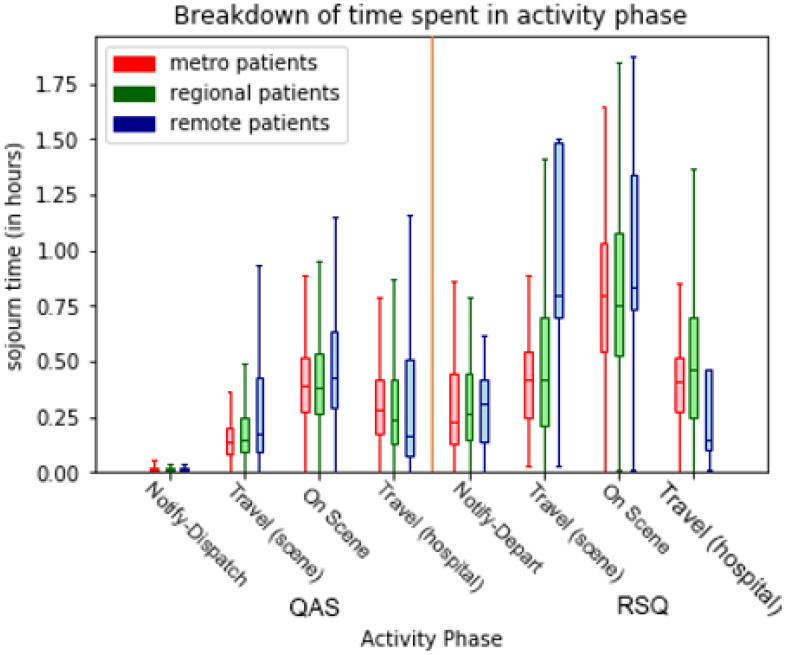
Comparison (by destination hospital remoteness) of transport segment durations—ground and air.

**Figure 14:**
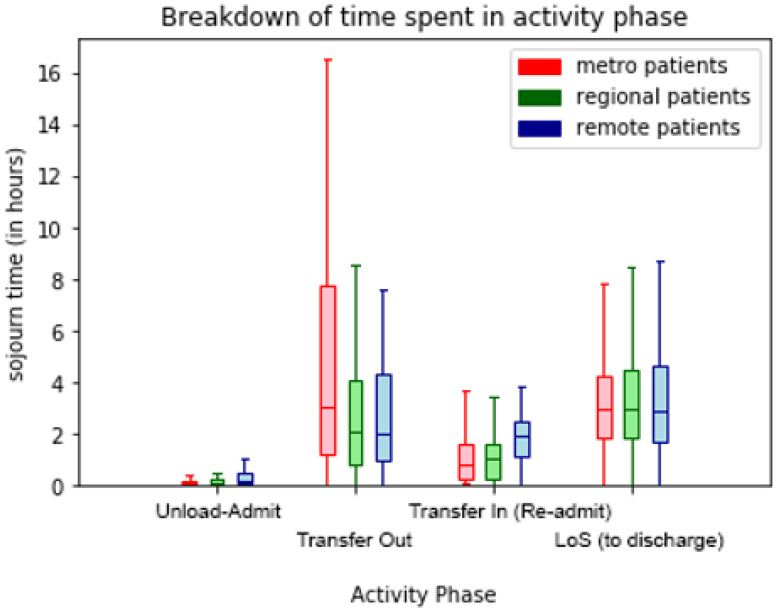
Comparison (by incident region) of hospital segment durations.

**Figure 15:**
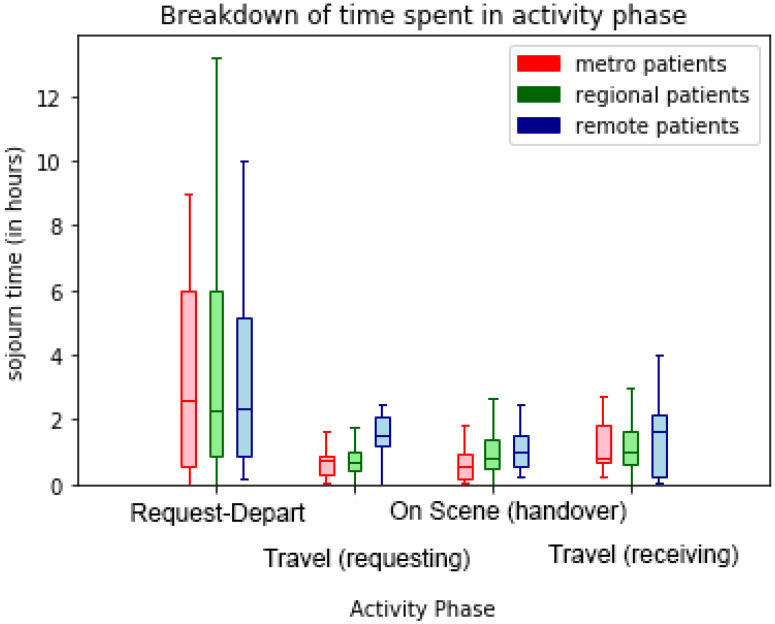
Comparison (by incident region) of inter-hospital transport segments durations.

The box-and-whisker plots of sojourn times for transport phases in Figure 13, Figure 14 and Figure 15 visualise the performance variations across process phases by quartile of the respective patient cohorts. In Figure 13 the effect of remoteness is clearly evident on travel times with remote patients involving longer travel-to/from-scene times (particularly for RSQ (air-ambulance) travel-to-scene times). Further differences are evident in the comparative on-scene times between ground (QAS) and air (RSQ) ambulance crews. This is likely reflective of the time taken to stabilise a patient prior to take-off (as some critical interventions are avoided once in the air), and the time required to appropriately package the patient for air travel.

### 5.4. Process Improvement Opportunities

Discussion of the study analysis results with process owners resulted in the following process improvement opportunities:

1. **Automated data collection—timestamped information** As this study, and others, has shown, event data quality, particularly relating to timestamps, impacts on the ability to apply process mining. While this study derived useful insights into the process, much effort was required to identify and deal with timestamp data quality. It is noted that much of the data is collected by people whose primary focus is patient welfare and not data entry with the result that there are many situations where data relating to patient retrieval, transport and pre-hospital care is entered manually (e.g., no aero-medical data is automatically captured). While some data points are collected automatically, it is recommended that options for automatic data collection be trialled. Options such as wearable technology and voice recognition would allow doctors and paramedics to record waypoints and interventions without interrupting treatment to patients. Options such as QR codes for equipment and drugs could be used to automatically record times and usage.
2. **On-scene patient identifier across attending crews**. Currently, each crew attending a patient completes a separate electronic Accident Report Form (eARF). The eARF records patient attributes such as gender and age (estimated age if not able to be directly confirmed) but, while each eARF contains an Incident identifier, there is no attribute that allows multiple eARFs for the same patient at the same incident to be easily retrospectively matched. In this study, of the 92,420 QAS CAD records (ground-based ambulances responding to a ‘000’ emergency call) 17,304 (18.7%) included an eARF that could not be linked to a patient. Further, of the 45,296 separate incidents (road traffic crashes), 8108 incidents included at least one patient that was retrospectively identified by the Data Linkage Unit (from data in the eEARF) and, at least one patient that was not able to be identified by the DLU from data in the eARF. Thus it is possible that information about an individual patient (observations and care given) recorded by different crews is not ultimately reconciled. This would be obviated by the use of an on-scene patient identifier.
3. **Automated assistance in determining transport destination** Currently, the transport destination for injured persons is usually determined by the on-scene paramedics based on their local knowledge of hospital and health facility locations, road and traffic conditions, etc. It is recommended to supplement this decision with automated support from systems that are aware of not only road and traffic conditions, but hospital conditions (e.g., bed availability).
4. **AI supported emergency call triage** The job of the emergency call centre operators is, on receipt of a ‘000’ emergency call, to determine an appropriate response. Callers to ‘000’ are rarely medically trained and often in a state of distress, thus adding to the difficulty and the time required for the operator to triage the call. For many conditions, e.g., cardiac arrest, response time is directly related to patient outcome (survival). AI systems such as Corti (https://corti.ai/) have been reported to be able to identify cardiac arrest from background sounds with 95% accuracy [24], faster and more accurately than human call takers [25]. It is suggested that such a system be trialled in the Queensland setting.

## 6. Discussion

This section synthesises key lessons learned based on the data-driven insights gained from this process mining study in the healthcare sector combined with the feedback from healthcare stakeholders throughout the project. These insights are aimed at the process mining research community and the advocates for process mining techniques within the healthcare sector.

**Variants are the norm in any healthcare process.** This study further reaffirms the fact that control-flow variations resulting in spaghetti-like process models are the norm for any healthcare process, even for a relatively straight-forward pre-hospital transport process. Thus, we should not rule out infrequent variants without considering underlying reasons for these exceptional behaviours. The act of simplifying such messy models using frequency-based noise filters may not be the best approach for healthcare processes. The stakeholder-guided simplification approach used in this study with the help of the Inductive visual Miner tool may provide a better outcome for healthcare processes.

**Stakeholder-guided process discovery techniques are useful for healthcare processes.** This study encountered issues with automatically discovered process models from the point of view of being readable (by both analysts and process owners). With the assistance of domain experts and using the Inductive visual Miner tool, we manually edit the underlying structure of the discovered models to arrive at models agreed on by stakeholders as being useful for analysis. This limitation of most existing automated discovery tools leads to the notion of tools that support guided discovery in which expert advice can be incorporated into discovery algorithms.

**Timestamps with different granularities can have a severe negative effect on the order of events.** An interesting feature of this case study is that we are looking at a process that has a very short duration (i.e., less than one hour in most cases) and therefore small differences in the recorded timestamps can result in incorrect ordering of events as seen in discovered models. In the emergency pre-hospital settings, things happen at a fast pace and the recording of some activities are optional and manual. Therefore it is expected that event ordering issues will be present in the log, which in turn, will lead to complexities in discovered process models.

**Multiple potential case notions can result in data correlation challenges.** Data correlation challenges associated with linking data from multiple parties were encountered as expected. The added complexity of linking multiple patients, multiple responding units and potential multiple transporting units (for example two transport units attended and one of these transported two patients while the other did not transport a patient) made the record linkage more complex. Therefore, it is essential that the preparation of an event log from a data set is closely guided by the key questions of interest, and thus an appropriate case notion is then determined while taking into account any data quality issues. New approaches to objectively assess the suitability of data attributes for process mining are required. Our recent work [26] describes RDB2Log which (i) uses metrics to quantitatively assess the quality of (relational) source data across 12 different quality dimensions, and (ii) uses the quality assessment to guide users in the semi-automated creation of event logs.

**Process mining uncovers the end-to-end patient journey in a healthcare setting.** Although a lot of data is being collected within the healthcare setting, stitching it all together to trace a patient’s journey through the different areas is generally challenging. This case study demonstrated how data from different sources can be identified and linked together to enable a ‘journey’ view of the patient through the healthcare system as well as identify appropriate data points to monitor clinical indicators and measure patient outcomes across an entire state.

**Prescriptive process models are needed to (automatically) generate process improvement recommendations from process mining insights.** Most process mining techniques are descriptive in nature, that is, they provide insights as to what has happened. They typically do not recommend actions to take in terms of how to improve these processes. Therefore, process improvement opportunities are largely generated by process owners in light of these process mining results. The field of process mining would be to move towards prescriptive (automatically derived) identification of improvement opportunities.

## 7. Conclusions

This case study examined some specific questions of interest to Queensland’s emergency services in relation to pre-hospital care and transport of persons injured in motor vehicle accidents. The study reported on challenges faced by the analysts in compiling an event log suitable for use in a process mining analysis from multiple disparate data sources, in particular, challenges in linking the different data sources to allow analysis at the level of individual patient journeys. Automated process discovery resulted in complex models, highlighting the variability in the retrieval and transport process. The discovered models were too complex to be useful requiring domain expert-guided editing to achieve usable models. Despite these models being deemed readable, representative of the overall process, and useful for analysis, they did remove many of the deviations, hence obfuscating potentially interesting process behaviours. Comparative process analysis highlighted performance differences relating to remoteness. Despite comprehensive analysis, the descriptive nature of process mining techniques (focusing on what has happened) meant that process improvement strategies were largely generated in concert with the process owners. This paper has presented methods for deriving event logs from multiple, disparate data sources, as well as approaches for process discovery and comparative analysis that are not limited to the pre-hospital setting, but are generalisable to other contexts. In particular, our approach would be applicable in contexts involving (i) multiple, distinct processes and data sources (from separate organisations), (ii) which have a somehow shared notion of case (iii) and which need to be combined to derive an over-arching, end-to-end process for analysis.

## Figures and Tables

**Table 1:** Summary of event log.

Attribute	Frequency	Attribute	Frequency
Number of events	366,754	Number of cases	42,603
Duration of cases (max)	8 days 4 h	Event per case (max, min)	45.3
Duration of cases (median)	50.9 min	Events per case (median)	7
Duration of cases (mean)	10.2 h	Events per case (mean)	8.6
Number of trace variants	2863		

**Table 2:** Event log and cohort summary.

Attribute	Log	Cohort 1	Cohort 2	Cohort 3
Number of cases	42,603	12,552	8231	21,820
Number of events	366,754	49,315	50,033	267,339
Duration of cases (max)	8 days 4 h	7 days 23 h	2 days 22 min	8 days 4 h
Duration of cases (mean)	10.2 h	16.2 min	68.2 min	19.2 h
Duration of cases (IQR)	5.0 h	3.75 h	35 min	11.4 h
Activities	49	5	16	49
Event per case (max)	45	7	25	45
Event per case (mean)	8.6	4	6	12.3
Number of trace variants	2969	43	57	2800

**Table 3:** Outcome by cohort as given by last recorded event.

**Cohort 1 Attend, no transport**		
**Activity (Org)**	**Cases**	**Cohort %**
AT_PATIENT (QAS)	8101	64.6%
ON_SCENE (QAS)	4398	35.1%
DISPATCH (QAS)	33	0.3%
**Cohort 2 Transport, no hospital**		
**Activity (Org)**	**Cases**	**Cohort %**
AT_DEST (QAS)	8058	98.2%
ARR_RECEIVING (RSQ)	99	1.2%
HOSPITAL_ADMIT (QHAPDC)	46	0.6%
**Cohort 3 Transport, hospital**		
**Activity (Org)**	**Cases**	**Cohort %**
ED_DISCHARGE	13,331	61.1%
HOSPITAL_DISCHARGE	7899	36.2%
HOSPITAL_ADMIT	134	0.6%
TRANSFER	92	0.4%
HOSPITAL_DEATH	53	0.25%
ED_DEATH	8	0.03%
OTHER	319	1.4%

## Appendix A. Interpretation of Activity Labels

**Table A1:** Activity labels and description.

**Activity (Source = QAS)**	**Interpretation**
D_RECEIVED	‘000’ emergency call received
D_DISPATCH	(Ground) ambulance dispatched to attend
D_ON_SCENE	(Ground) ambulance arrives at (or as close as it is possible to get the vehicle) to the incident scene
D_AT_PATIENT	Paramedics arrive at the patient
D_DEPART_SCENE	(Ground) ambulance leaves the incident scene with a patient
D_AT_DEST	(Ground) ambulance arrives at destination (hospital, health facility, handover point, airport, etc.)
**Activity (Source = RSQ)**	**Interpretation**
TEAM_ACTIVATED	Following request to launch, the medical time to fly is assembled
READY_TO_DEPART	(Air) ambulance is prepped and ready to fly
DEPART_WITH_MEDICAL_TEAM	(Air) ambulance leaves en route to patient pick-up location
LAND_AT_DESTINATION	Land as close as possible to patient pick-up point
AT_SCENE_PATIENT	Doctor/paramedic arrive at the patient
DEPARTURE_READY	Patient is stabilised and loaded on the (air) ambulance
ACTUAL_TIME_DEPART	(Air) ambulance departs pick-up point with patient
ARRIVE_AT_RECEIVING_HOSPITAL	(Air) ambulance arrives at hospital
DEPART_RECEIVING_HOSPITAL	(Air) ambulance departs hospital (on return leg)
ARRIVE_BACK_AT_BASE	(Air) ambulance arrives back at base (for re-tasking)
**Activity (Source = ED − QHAPDC)**	**Interpretation**
ED_ADMIT_TO_[TSL]	Patient admitted to emergency department of trauma service level: TSL = MAJOR, REGIONAL, or HOSPITAL
ED_TRANSFER_FROM_[TSL]	Patient admitted to emergency department after being transferred from another hospital with trauma service level: TSL = MAJOR, REGIONAL, or HOSPITAL
ED_DISCHARGE_FROM_[TSL]	Patient discharged from emergency department of trauma service level: TSL = MAJOR, REGIONAL, or HOSPITAL
ED_TRANSFER_TO_[TSL]	Patient discharged from emergency department and transferred to another hospital with trauma service level: TSL = MAJOR, REGIONAL, or HOSPITAL
ED_PHYSICALLY_LEAVE	Patient physically leaves the emergency department
ED_DEATH	Patient died in hospital ward
HOSPITAL_ADMIT_TO_[TSL]	Patient admitted to hospital ward of trauma service level: TSL = MAJOR, REGIONAL, or HOSPITAL
HOSPITAL_TRANSFER_FROM_[TSL]	Patient admitted to hospital ward after being transferred from another hospital with trauma service level: TSL = MAJOR, REGIONAL, or HOSPITAL
HOSPITAL_DISCHARGE_FROM_[TSL]	Patient discharged from hospital ward of trauma service level: TSL = MAJOR, REGIONAL, or HOSPITAL
HOSPITAL_TRANSFER_TO_[TSL]	Patient discharged from hospital ward and transferred to another hospital with trauma service level: TSL = MAJOR, REGIONAL, or HOSPITAL
HOSPITAL_DEATH	Patient died in emergency department
